# The mPINC survey: Impacting US maternity care practices

**DOI:** 10.1111/mcn.13092

**Published:** 2020-11-04

**Authors:** Jennifer M. Nelson, Daurice A. Grossniklaus, Deborah A. Galuska, Cria G. Perrine

**Affiliations:** ^1^ Division of Nutrition, Physical Activity, and Obesity National Center for Chronic Disease Prevention and Health Promotion, Centers for Disease Control and Prevention Atlanta Georgia USA; ^2^ United States Public Health Service Commissioned Corps Washington District of Columbia USA

**Keywords:** breastfeeding, maternity practices, mPINC survey

## Abstract

The Centers for Disease Control and Prevention administered the original Maternity Practices in Infant Nutrition and Care (mPINC) survey, a census of all US birth facilities, from 2007 to 2015 to monitor infant feeding‐related maternity care practices and policies. The purpose of this paper is to describe the many uses of mPINC data. Hospitals, organizations and governments (federal, state and local) have used the mPINC survey as a tool for improving care among the populations they serve. Nationally, the mPINC survey has been used to document marked improvements in infant feeding‐related maternity care. Researchers have used the mPINC data to examine a variety of questions related to maternity care practices and policies. The newly revised mPINC survey (2018) has been designed to capture changes that have occurred over the past decade in infant feeding‐related US maternity care. Hospitals, organizations, governments and researchers will be able to continue using this important tool in their efforts to ensure US maternity care practices and policies are fully supportive of breastfeeding.

**Key messages**

- The mPINC survey, a biennial census of US maternity care facilities, has been used for surveillance, quality improvement and research.
- Data from the mPINC survey have captured improvements in US maternity care practices and policies.
- The mPINC survey has been a valuable tool for hospitals, organizations, governments and researchers to improve breastfeeding support provided to mothers and infants.

## 1 INTRODUCTION

Breastfeeding is considered the optimal feeding method for most infants (American Academy of Pediatrics: Section on Breastfeeding, 2012) and reduces chronic diseases in women (Feltner et al., 2018). Evidence‐based maternity care practices are known to improve breastfeeding exclusivity (Perrine, Scanlon, Li, Odom, & Grummer‐Strawn, 2012) and duration (DiGirolamo, Grummer‐ Strawn, & Fein, 2008). The first hospital in the United States designated as part of the Baby‐Friendly Hospital Initiative, a programme designed to recognize hospitals offering evidence‐based maternity care as outlined in the Ten Steps to Successful Breastfeeding (Ten Steps), was in 1996, but widespread participation in the initiative was low for many years (Baby‐Friendly USA, 2016). Although local (Kovach, 2002) and state (Rosenberg, Stull, Adler, Kasehagen, & Crivelli‐Kovach, 2008) efforts had been made to assess hospital practices supportive of breastfeeding, there were no national or regional data on implementation of these practices despite the critical role hospitals were known to play in supporting breastfeeding. Thus, in the fall of 2003, the Centers for Disease Control and Prevention (CDC) hosted a meeting of experts to explore the feasibility of national surveillance of maternity care practices related to breastfeeding, including data needs, potential barriers to data collection and potential methods for surveillance. A 3‐year development phase began that culminated with the implementation of the first Maternity Practices in Infant Nutrition and Care (mPINC) survey in 2007. The original mPINC survey, covering seven domains of maternity care (Labor and Delivery, Feeding of Breastfed Infants, Breastfeeding Assistance, Mother‐Infant Contact, Discharge Care, Staff Training and Structural and Organizational Aspects of Care Delivery), was administered every 2 years until 2015 (Perrine et al., 2015). The domains of care were scored from 0 to 100 to generate seven subdomain scores, which were averaged to calculate a total mPINC score. Both the mPINC survey and scoring algorithm are available by emailing: mpinc@cdc.gov.

All hospitals and free‐standing birth centres in the United States and Territories (American Samoa, Guam, Northern Mariana Islands, Puerto Rico and the US Virgin Islands) (hereafter, “states” unless otherwise noted) with maternity beds in the year prior to the survey were eligible to participate. The main facility switchboard was contacted with a request to speak with the manager of the Mother‐Baby unit. Once reached, this manager confirmed the hospital's eligibility and identified the most appropriate person to receive the survey. Hospitals were encouraged to get input from key staff, as needed, when completing the survey. Completed surveys were submitted either electronically or via paper with response rates ranging from 82% to 83% for all five cycles (range of participating facilities: 2,582–2,742). Given the high response rate of this census, data on maternity practices and policies covering 74% to 82% of US births were obtained (Table 1). The percent of surveys being completed by more than one individual ranged from 39% in 2007 to 75% in 2015. The number of individuals contributing to a survey in 2015 ranged from 1 to 16, with a median of 3. Across survey years, some of the most common titles of individuals contributing to survey responses included: Mother Baby Unit Manager/Supervisor, Labour and Delivery Unit Manager/Supervisor, Lactation Services Coordinator, Lactation Consultant/Specialist and Maternity Care Services Director/Manager.

**TABLE 1:** Approximate number and proportion of US births covered by the mPINC survey, 2007–2015

Survey year	Number of facilities	Estimated births captured by mPINC	Estimated US births (Martin, Hamilton, Osterman, Driscoll, & Mathews, 2017)	Estimated proportion of US births captured by mPINC (%)
2007	2,676	3,202,843	4,316,233	74
2009	2,651	3,138,598	4,130,665	76
2011	2,730	3,225,940	3,953,590	82
2013	2,648	3,138,622	3,932,181	80
2015	2,558	3,066,316	3,978,497	77

*Note*: Data, including number of facilities and estimated births captured by mPINC, from the US Territories (American Samoa, Guam, Northern Mariana Islands, Puerto Rico, and the US Virgin Islands), were not included.

Abbreviation: mPINC, Maternity Practices in Infant Nutrition and Care.

The purpose of this paper is to describe the many uses of mPINC data, including for national and state surveillance of infant feeding‐related maternity care practices, for hospital quality improvement, and for public health research.

### 1.1 mPINC: Data for national surveillance

**FIGURE 1:**
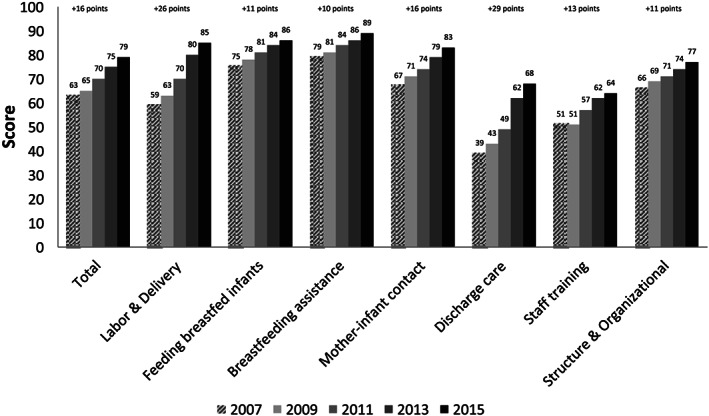
Average total and subdomain scores from CDC's Maternity Practices in Infant Nutrition and Care (mPINC) survey, 2007–2015

**FIGURE 2:**
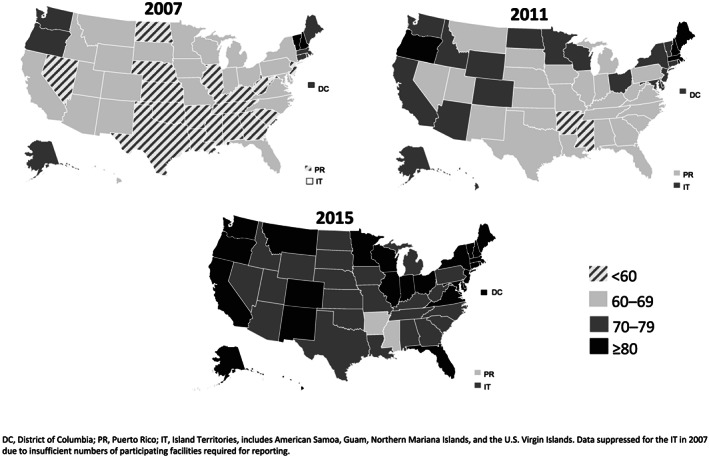
Average total score from CDC's Maternity Practices in Infant Nutrition and Care (mPINC) survey, by state, 2007, 2011 and 2015

Nationally, the total mPINC score increased from 63 in 2007 to 79 in 2015, a 16‐point increase (Figure 1). Subdomain scores for each area of care also increased across the time period (range: +10 to +29 points). The largest increases were seen in Discharge Care (+29 points), influenced by the decline in distribution of discharge bags containing infant formula (Nelson, Li, & Perrine, 2015), and Labour and Delivery (+26 points), influenced by the increase in skin‐to‐skin practices (Boundy, Perrine, Barrera, Li, & Hamner, 2018). All states and Territories increased their mPINC total scores between 2007 and 2015. Of 56 states and Territories covered by the mPINC survey, in 2007, 18 states, predominantly in the south and middle of the country, had an average total mPINC score <60, whereas only 2 states, New Hampshire and Vermont, had an average total score ≥80 (Figure 2). By 2015, this pattern had improved, with no states having an average total score <60 and 26 states having an average total score ≥80.

Aggregate, national mPINC data have been used extensively to describe US maternity care practices and policies. For example, analyses have examined use of human milk in neonatal intensive care units (Boundy, Perrine, Nelson, & Hamner, 2017; Perrin, 2018; Perrine & Scanlon, 2013), monitored progress towards the implementation of the Ten Steps (Barrera, Nelson, Boundy, & Perrine, 2018; Bartick, Edwards, Walker, & Jenkins, 2010; Beauregard, Nelson, & Hamner, 2018; Boundy et al., 2018; Centers for Disease & Prevention, 2011; Grossniklaus et al., 2017; Nelson, Perrine, Scanlon, & Li, 2016; Perrine et al., 2015) and compliance with the International Code of Marketing of Breast‐milk Substitutes (Nelson et al., 2015), and described employee lactation support services (Allen, Belay, & Perrine, 2014). Further, mPINC data have been used by the United Health Foundation to assess the health of women, infants and children at the state and national level (United Health Foundation, 2016).

Additionally, mPINC data have been used to facilitate responses to public health emergencies. During the 2009 H1N1 pandemic, mPINC data about the prevalence and locations of facilities with separate newborn nurseries and/or rooming‐in for mothers and infants during the birth hospitalization informed CDC’s development of H1N1 guidance to help US hospitals continue safely caring for mothers and infants. More recently, during the early phases of the COVID‐19 pandemic, routine maternity care practices (e.g., maternal–infant rooming‐in) were disrupted over concerns of SARS‐CoV‐2 transmission from an infected mother to her newborn. A supplemental questionnaire was quickly developed and is currently being administered to participating mPINC hospitals in order to understand how the pandemic has influenced hospital practices.

### 1.2 mPINC: Data for individual hospital improvement

Each facility that participated in the mPINC survey received six copies of an individualized Benchmark Report, which was sent to the person who originally received the survey as well as to key leadership positions including: Chief Executive Officer, Director of Hospital Quality Improvement, Obstetrics Medical Director, Paediatrics Medical Director and Nurse Manager for Mother‐Baby Services. These Benchmark Reports highlight strengths of the facility as well as areas where improvements may be needed. In the report, the facility's total and subdomain scores were provided and compared with three groups: facilities of similar size, facilities within their state and all US facilities.

Facilities have used the subdomain scores and percentile rankings in the Benchmark Reports to identify areas to improve the maternity care they provide. Two examples are from safety‐net hospitals whose patients come from low‐income, medically underserved, minority populations. The University Medical Center of El Paso, located in a remote area along the Texas‐Mexico border, used the mPINC survey and other resources [e.g., Baby‐Friendly Hospital Initiative guidelines (Baby‐Friendly USA, 2016)] to guide implementation of improvements in their maternity care and to serve as an external benchmark for monitoring these changes (Eganhouse, Gutierrez, Cuellar, & Velasquez, 2016). One change was the creation of a paper record, which included components of the mPINC data (e.g., initiation of breastfeeding), used to improve patient care handoffs when mothers were transferred from Labour and Delivery to the Mother‐Baby unit. This change, in addition to others, led to the hospital increasing their mPINC total score from 65 (2011) to 92 (2013) and their exclusive breastfeeding rate from 16% (2012) to 42% (2015). Boston Medical Center used their mPINC scores to identify areas in their maternity care practices where changes could improve breastfeeding support (Preer, Pisegna, Cook, Henri, & Philipp, 2013). Aiming to decrease maternal–infant separation and to increase exclusive breastfeeding, delaying the newborn bath was identified as an intervention to achieve both goals. As such, newborn baths were delayed from an average of 2.4 h of life (preintervention) to an average of 13.5 h (postintervention). Compared with infants born before the intervention, infants born after the intervention had 166% greater odds of initiating breastfeeding, 39% greater odds of breastfeeding exclusively and 59% greater odds of near‐exclusive breastfeeding, as defined by having 90% to <100% breast milk feeds.

A report by the National Institute for Children's Health Quality (NICHQ) (2015) described a survey conducted by the Indiana Perinatal Network that found many Indiana hospitals preferred using their mPINC data to facilitate quality improvement, whereas others were interested in pursuing Baby‐Friendly designation to facilitate improvement. Labbok, Taylor, & Nickel (2013) used the mPINC survey as a measure of hospitals' breastfeeding support to help understand barriers to implementation of the Ten Steps faced by hospitals serving low wealth populations. Additionally, hospitals often communicate their mPINC scores to their patients and communities to celebrate their success in providing quality maternity care (Massachusetts General Hospital, n.d.; Cheyenne Regional Medical Center, n.d.; Adventist Health, 2017; Beebe Healthcare, 2017; Brookings Health System, 2017; Heart of the Rockies Regional Medical Center, 2016; Henry Community Health, 2017; South Peninsula Hospital, 2016).

### 1.3 mPINC: Data used by states, counties and other organizations

As with national data, states and other organizations have used mPINC data in a variety of ways to improve maternity care within their populations. States such as California (California Department of Public Health, 2017), Michigan (Michigan Department of Health and Human Services, 2017) and Minnesota (Minnesota Department of Health, 2018) have used their state‐specific mPINC data to develop more targeted data reports. For example, the California Department of Public Health created region‐level mPINC benchmark reports (California Department of Public Health, 2017). Similarly, the Minnesota Department of Health's Special Supplemental Nutrition Program for Women, Infants and Children (WIC) created region‐specific mPINC scores, allowing a better understanding of regional differences in hospital practices (Minnesota Department of Health, 2018).

There are many state‐specific recognition programmes designed to acknowledge hospitals for implementing a portion or all of the Ten Steps. Based on our work with state health departments, breastfeeding coalitions and other stakeholders, we are aware there are at least five states that use state‐specific mPINC data, along with other breastfeeding data, to inform their recognition program [Alabama (Alabama Breastfeeding Committee, n.d.), Kansas (High 5 for Mom & Baby, n.d.), Missouri (Missouri Department of Health & Senior Services, n.d.), Pennsylvania (Pennsylvania Department of Health, n.d.) and Texas (Texas Department of State Health Services, 2012)], including reporting of mPINC participation and/or total score. For example, the Kansas Breastfeeding Workgroup has used Kansas' mPINC data to monitor progress towards implementation of their *High 5 for Mom and Baby* programme, which helps hospitals adopt five evidence‐based, breastfeeding‐related maternity practices (High 5 for Mom & Baby, n.d.).

Also, based on our work with breastfeeding stakeholders, we are aware of at least four states [Hawaii (Kahin et al., 2017), Maryland (Maryland Department of Health and Mental Hygiene, 2012), Nebraska (Nebraska Department of Health and Human Services, 2015) and Oregon (Oregon Health Authority, 2017)] that have used their mPINC data to inform or highlight their breastfeeding work. For example, the Hawaii State Department of Health's Healthy Hawaii Initiative was able to monitor progress of the Baby‐Friendly Hawaii project, which aimed to improve evidence‐based maternity care practices and in‐hospital exclusive breastfeeding rates, by monitoring preproject and postproject mPINC scores (Kahin et al., 2017). Further, at least 12 states disseminate their state‐specific mPINC report and/or link to the mPINC website via the state's or breastfeeding coalition's website.

The mPINC data have played a role in state‐ and county‐based quality improvement initiatives in places such as Iowa (Stockton, n.d.; Lillehoj & Dobson, 2012), Massachusetts (Edwards & Philipp, 2010), Puerto Rico (Piovanetti, Calderon, & Castaner, 2015), Tennessee (Healthy Tennessee Babies, n.d.) and Whatcom County, Washington (Whatcom County Health Department, 2011). An example of states working with individual hospitals is the Iowa Department of Public Health (IDPH) that realized, prompted by the state's mPINC ranking, that women delivering in rural hospitals did not have the same access to breastfeeding education and support compared with other women (Stockton, n.d.). In turn, it set up breastfeeding education training targeted at staff of rural hospitals with a significant number of Medicaid births, resulting in a majority of hospitals implementing at least three of the Ten Steps. To further this effort, IDPH offered targeted technical assistance to select hospitals which were rural, had high Medicaid birth rates and had mPINC scores lower than Iowa's total score in an effort to identify where improvements could be made. An example of state‐level quality improvement is how Healthy Tennessee Babies developed a breastfeeding toolkit, in which resources to help hospitals improve their breastfeeding practices were organized by the mPINC domains of care (Healthy Tennessee Babies, n.d.). In addition, NICHQ, as part of the Indiana State Department of Health (ISDH)/NICHQ Partnership for Breastfeeding Improvement, facilitated a meeting of experts aimed at discussing the current status of breastfeeding in Indiana and guiding ISDH's future breastfeeding work (National Institute for Children's Health Quality, 2015). These experts identified mPINC regional training and technical assistance, along with hospital visits, as a current strength in intrapartum care being provided in Indiana.

States and organizations have also used the mPINC survey as a basis for [Minnesota Breastfeeding Coalition (Minnesota Breastfeeding Coalition, 2016), New Mexico Breastfeeding Taskforce (RWJF Center for Health Policy at the University of New Mexico, 2016)] or in comparison with [New York (Dennison, Nguyen, Gregg, Fan, & Xu, 2016)] their own hospital assessment tools. The New York State Department of Health conducted their own maternity care practices surveys in 2009 and 2014 (Dennison et al., 2016). Though there were slight differences between the surveys (e.g., an 84% response rate to mPINC from New York hospitals vs. a 100% response rate to the state survey), both surveys were able to measure a consistent increase in the state‐wide composite score, demonstrating improved maternity practices. The New Mexico Breastfeeding Taskforce created their own hospital‐specific report cards which, similar to the mPINC Benchmark Report, provided hospitals with a comparison of their breastfeeding performance to all New Mexico facilities and facilities of similar size (RWJF Center for Health Policy at the University of New Mexico, 2016).

### 1.4 mPINC: Data for public health research

In addition to the uses described above, mPINC data have also been used for public health research. For example, several studies have linked mPINC scores with breastfeeding data from other sources and have found breastfeeding outcomes were positively correlated with mPINC scores. Two studies (Barrera, Beauregard, Nelson, & Perrine, 2019; Patterson, Keuler, & Olson, 2018) linked mPINC data to in‐hospital exclusive breastfeeding rates collected by The Joint Commission, finding that hospitals with higher mPINC scores had higher in‐hospital exclusive breastfeeding rates. Self‐reported maternal data from the Pregnancy Risk Assessment Monitoring System were linked with mPINC to demonstrate that women who delivered in hospitals with higher mPINC scores were more likely to be breastfeeding and to be exclusively breastfeeding at 8 weeks postpartum (Nelson et al., 2018). The mPINC data from 48 hospitals in Alabama were linked with infant feeding data from the newborn screening database, finding infants delivered in hospitals, which had Structural and Organizational Aspects of Care Delivery, a mPINC subdomain, scores higher than the state mean were more likely to be breastfeeding (Li et al., 2014).

### 1.5 Limitations

Despite the benefits of the mPINC survey, it does have certain limitations. The mPINC data are self‐reported by hospital staff. Although CDC specifically asks for the contact information of the person with the most knowledge about these practices to send the survey to, and encourages input of other key staff, the validity and reliability of these data have not been formally evaluated nor has the scoring algorithm. As described above, studies have shown that higher mPINC scores are associated with exclusive breastfeeding at hospital discharge (Barrera et al., 2019) and any breastfeeding at 8 weeks (Nelson et al., 2018), suggesting that the data captured by mPINC are reflective of practice. Another limitation is that very little demographic information on the hospital, including on the population served, is collected. Having this information would allow for better understanding of health disparities in maternity care provided. Efforts, albeit with limitations, have been made to address this by linking the mPINC data to demographic data collected from the US census (Boundy et al., 2017; Lind, Perrine, Li, Scanlon, & Grummer‐Strawn, 2014).

### 1.6 Future directions

Action 7 of the Surgeon General's *Call to Action to Support Breastfeeding* is to ensure US maternity care practices are fully supportive of breastfeeding (US Department of Health and Human Services, 2011). The mPINC survey, used as a surveillance tool, monitors this action. US maternity care practices continue to evolve as we seek to provide optimal care for mothers and their infants. Examples of some changes include caring for an increasing number of infants born with Neonatal Abstinence Syndrome (NAS) (Ko et al., 2016) who benefit from breastfeeding (Edwards & Brown, 2016) and rooming‐in (MacMillan et al., 2018), increasing attention about how to safely implement practices such as skin‐to‐skin care (Feldman‐Winter, Goldsmith, Committee On the Fetus and Newborn, & Task Force On Sudden Infant Death Syndrome, 2016) and an update of international guidance on breastfeeding‐supportive practices (World Health Organization, 2018).

Given these marked changes in maternity care practices, a revision of the mPINC survey was judged necessary. An expert panel was convened in 2014 to provide input into revising the survey. CDC took this feedback, along with input from additional subject matter experts, and revised both the survey methods and the questionnaire. The survey was then open for public comment as required by government administrative processes. In November 2018, the new mPINC survey was launched.

The survey's focus remains to provide hospitals with specific feedback to provide national and state surveillance on maternity care practices, to inform quality improvement efforts and to help researchers gain a better understanding of US maternity care practices. New or expanded topics include use of donor breast milk, delayed umbilical cord clamping, selected safety practices and care of infants with NAS. Given these expanded topics, mPINC data on the care of the infant diagnosed with NAS have been proposed as one indicator to monitor progress on the implementation of the Protecting Our Infants Act (Substance Abuse and Mental Health Services Administration, 2019), a law designed to address problems stemming from prenatal opioid exposure. Because all survey questions were updated, scores from the original survey (2007–2015) are not comparable with scores from the revised survey (2018).

## 2 CONCLUSION

The mPINC survey has been a valuable surveillance, quality improvement and research tool in tracking and helping improve maternity care practices, and policies in the US. Facilities have used their facility‐specific Benchmark Reports to identify potential strengths and weakness in the care they provide to mothers and newborns. States and organizations have used state‐specific data to make programmatic decisions to support breastfeeding among their constituents. National, aggregate data have captured marked improvements in infant feeding‐related maternity care practices and policies in the United States. We anticipate that the new mPINC survey will continue to be an impactful tool for hospitals, organizations, governments and researchers in their efforts to ensure US maternity care practices and policies are fully supportive of breastfeeding.

## CONFLICT OF INTEREST

The authors declare that they have no conflicts of interest.

## CONTRIBUTIONS

JN, DG, DG and CP contributed to the analysis and/or interpretation of data as well as reviewing and revising the manuscript. All authors approved the final manuscript as submitted and agree to be accountable for all aspects of the work. The findings and conclusions in this report are those of the authors and do not necessarily represent the official position of the Centers for Disease Control and Prevention.

## References

[mcn13092-bib-0001] Adventist Health . (2017). Lodi health receives standout score for maternity care. Retrieved from https://www.adventisthealth.org/blog/2017/february/lodi-health-receives-standout-score-for-maternit/

[mcn13092-bib-0002] Alabama Breastfeeding Committee . (n.d.). Better Bama babies hospital recognition program. Retrieved from http://alabamabreastfeeding.org/bbb/

[mcn13092-bib-0003] Allen, J. A. , Belay, B. , & Perrine, C. G. (2014). Using mPINC data to measure breastfeeding support for hospital employees. Journal of Human Lactation, 30(1), 97–101. 10.1177/0890334413495974 23860266 PMC4516120

[mcn13092-bib-0004] American Academy of Pediatrics: Section on Breastfeeding . (2012). Breastfeeding and the use of human milk. Pediatrics, 129(3), e827–e841. 10.1542/peds.2011-3552 22371471

[mcn13092-bib-0005] Baby‐Friendly USA . (2016). The guidelines and evaluation criteria. Retrieved from https://www.babyfriendlyusa.org/for-facilities/practice-guidelines/

[mcn13092-bib-0006] Barrera, C. M. , Beauregard, J. L. , Nelson, J. M. , & Perrine, C. G. (2019). Association of maternity care practices and policies with in‐hospital exclusive breastfeeding in the United States. Breastfeeding Medicine, 14(4), 243–248. 10.1089/bfm.2018.0196 30807205 PMC6681453

[mcn13092-bib-0007] Barrera, C. M. , Nelson, J. M. , Boundy, E. O. , & Perrine, C. G. (2018). Trends in rooming‐in practices among hospitals in the United States, 2007‐2015. Birth, 45, 432–439. 10.1111/birt.12359 29806099 PMC6235708

[mcn13092-bib-0008] Bartick, M. , Edwards, R. A. , Walker, M. , & Jenkins, L. (2010). The Massachusetts baby‐friendly collaborative: Lessons learned from an innovation to foster implementation of best practices. Journal of Human Lactation, 26(4), 405–411. 10.1177/0890334410379797 20876344

[mcn13092-bib-0009] Beauregard, J. L. , Nelson, J. M. , & Hamner, H. C. (2018). Maternity care hospital trends in providing postdischarge breastfeeding supports to new mothers‐United States, 2007‐2015. Birth, 46, 318–325. 10.1111/birt.12408 30402907

[mcn13092-bib-0010] Beebe Healthcare . (2017). Beebe healthcare ranks among best in the nation for maternity care. Retrieved from https://www.beebehealthcare.org/news/awards-accreditations-accolades/beebe-healthcare-ranks-among-best-nation-maternity-care

[mcn13092-bib-0011] Boundy, E. O. , Perrine, C. G. , Barrera, C. M. , Li, R. , & Hamner, H. C. (2018). Trends in maternity care practice skin‐to‐skin contact indicators: United States, 2007‐2015. Breastfeeding Medicine, 13(5), 381–387. 10.1089/bfm.2018.0035 29782185 PMC9244860

[mcn13092-bib-0012] Boundy, E. O. , Perrine, C. G. , Nelson, J. M. , & Hamner, H. C. (2017). Disparities in hospital‐reported breast milk use in neonatal intensive care inits—United States, 2015. MMWR Morb Mortal Wkly Rep, 66(48), 1313–1317. 10.15585/mmwr.mm6648a1 29216029 PMC5757635

[mcn13092-bib-0013] Brookings Health System . (2017). 2016 an award‐winning year for Brookings health system. Retrieved from https://www.brookingshealth.org/news-events/news/2016-award-winning-year-brookings-health-system

[mcn13092-bib-0014] California Department of Public Health . (2017). Data to monitor progress in hospital policies and practices that support breastfeeding. Retrieved from https://www.cdph.ca.gov/Programs/CFH/DMCAH/CDPH%20Document%20Library/BFP/BFP‐Data‐InHospital‐ReleaseLetter‐2016‐Attachment.pdf

[mcn13092-bib-0015] Centers for Disease Control and Prevention . (2011). Vital signs: Hospital practices to support breastfeeding—United States, 2007 and 2009. MMWR. Morbidity and Mortality Weekly Report, 60(30), 1020–1025. 21814166

[mcn13092-bib-0016] Cheyenne Regional Medical Center . (n.d.). Centers for disease control and prevention's (CDC) quality benchmark report (2016). Retrieved from https://www.cheyenneregional.org/awards/maternity-care-centers-disease-control-preventions-cdc-quality-benchmark-report/

[mcn13092-bib-0017] Dennison, B. A. , Nguyen, T. Q. , Gregg, D. J. , Fan, W. , & Xu, C. (2016). The impact of hospital resources and availability of professional lactation support on maternity care: Results of breastfeeding surveys 2009‐2014. Breastfeeding Medicine, 11, 479–486. 10.1089/bfm.2016.0072 27644007

[mcn13092-bib-0018] DiGirolamo, A. M. , Grummer‐Strawn, L. M. , & Fein, S. B. (2008). Effect of maternity‐care practices on breastfeeding. Pediatrics, 122(Suppl 2), S43–S49. 10.1542/peds.2008-1315e 18829830

[mcn13092-bib-0019] Edwards, L. , & Brown, L. F. (2016). Nonpharmacologic management of neonatal abstinence syndrome: An integrative review. Neonatal Network, 35(5), 305–313. 10.1891/0730-0832.35.5.305 27636695

[mcn13092-bib-0020] Edwards, R. A. , & Philipp, B. L. (2010). Using maternity practices in infant nutrition and care (mPINC) survey results as a catalyst for change. Journal of Human Lactation, 26(4), 399–404. 10.1177/0890334410371212 20876345

[mcn13092-bib-0021] Eganhouse, D. J. , Gutierrez, L. , Cuellar, L. , & Velasquez, C. (2016). Becoming baby‐friendly and transforming maternity care in a safety‐net hospital on the Texas‐Mexico border. Nursing for Women's Health, 20(4), 378–390. 10.1016/j.nwh.2016.07.005 27520602

[mcn13092-bib-0022] Feldman‐Winter, L. , Goldsmith, J. P. , Committee On the Fetus and Newborn , & Task Force On Sudden Infant Death Syndrome . (2016). Safe sleep and skin‐to‐skin care in the neonatal period for healthy term newborns. Pediatrics, 138(3), e20161889 10.1542/peds.2016-1889 27550975

[mcn13092-bib-0023] Feltner, C. , Weber, R. P. , Stuebe, A. , Grodensky, C. A. , Orr, C. , & Viswanathan, M. (2018). Breastfeeding programs and policies, breastfeeding uptake, and maternal health outcomes in developed countries. Retrieved from https://effectivehealthcare.ahrq.gov/products/breastfeeding/research 30204377

[mcn13092-bib-0024] Grossniklaus, D. A. , Perrine, C. G. , MacGowan, C. , Scanlon, K. S. , Shealy, K. R. , Murphy, P. , … Grummer‐Strawn, L. M. (2017). Participation in a quality improvement collaborative and change in maternity care practices. The Journal of Perinatal Education, 26(3), 136–143. 10.1891/1058-1243.26.3.136 30723377 PMC6354630

[mcn13092-bib-0025] Healthy Tennessee Babies . (n.d.). Welcome to the TN breastfeeding toolkit! Retrieved from http://www.healthytennesseebabies.com/toolkit.aspx

[mcn13092-bib-0026] Heart of the Rockies Regional Medical Center . (2016). HRRMC family birthing center receives high marks in CDC survey. Retrieved from https://www.hrrmc.com/News/2016/November/HRRMC-Family-Birthing-Center-Receives-High-Marks.aspx

[mcn13092-bib-0027] Henry Community Health . (2017). BirthCare center at Henry community health receives national recognition. Retrieved from https://www.hchcares.org/birthcare-center-at-henry-community-health-receives-national-recognition/

[mcn13092-bib-0028] High 5 for Mom & Baby . (n.d.). The crucial role of hospitals. Retrieved from http://www.high5kansas.org/role-of-hospitals-breastfeeding.html

[mcn13092-bib-0029] Kahin, S. A. , McGurk, M. , Hansen‐Smith, H. , West, M. , Li, R. , & Melcher, C. L. (2017). Key program findings and insights from the baby‐friendly Hawaii project. Journal of Human Lactation, 33(2), 409–414. 10.1177/0890334416683675 28135119 PMC5499148

[mcn13092-bib-0030] Ko, J. Y. , Patrick, S. W. , Tong, V. T. , Patel, R. , Lind, J. N. , & Barfield, W. D. (2016). Incidence of neonatal abstinence syndrome—28 States, 1999‐2013. MMWR Morb Mortal Wkly Rep, 65(31), 799–802. 10.15585/mmwr.mm6531a2 27513154

[mcn13092-bib-0031] Kovach, A. C. (2002). A 5‐year follow‐up study of hospital breastfeeding policies in the Philadelphia area: A comparison with the ten steps. Journal of Human Lactation, 18(2), 144–154. 10.1177/089033440201800206 12033076

[mcn13092-bib-0032] Labbok, M. H. , Taylor, E. C. , & Nickel, N. C. (2013). Implementing the ten steps to successful breastfeeding in multiple hospitals serving low‐wealth patients in the US: Innovative research design and baseline findings. International Breastfeeding Journal, 8(1), 5 10.1186/1746-4358-8-5 23688264 PMC3669017

[mcn13092-bib-0033] Li, C. M. , Li, R. , Ashley, C. G. , Smiley, J. M. , Cohen, J. H. , & Dee, D. L. (2014). Associations of hospital staff training and policies with early breastfeeding practices. Journal of Human Lactation, 30(1), 88–96. 10.1177/0890334413484551 23603574

[mcn13092-bib-0034] Lillehoj, C. J. , & Dobson, B. L. (2012). Implementation of the baby‐friendly hospital initiative steps in Iowa hospitals. Journal of Obstetric, Gynecologic, and Neonatal Nursing, 41(6), 717–727. 10.1111/j.1552-6909.2012.01411.x 23030657

[mcn13092-bib-0035] Lind, J. N. , Perrine, C. G. , Li, R. , Scanlon, K. S. , & Grummer‐Strawn, L. M. (2014). Racial disparities in access to maternity care practices that support breastfeeding—United States, 2011. MMWR. Morbidity and Mortality Weekly Report, 63(33), 725–728. https://www.cdc.gov/mmwr/preview/mmwrhtml/mm6333a2.htm 25144543 PMC5779438

[mcn13092-bib-0036] MacMillan, K. D. L. , Rendon, C. P. , Verma, K. , Riblet, N. , Washer, D. B. , & Volpe Holmes, A. (2018). Association of rooming‐in with outcomes for neonatal abstinence syndrome: A systematic review and meta‐analysis. JAMA Pediatrics, 172(4), 345–351. 10.1001/jamapediatrics.2017.5195 29404599 PMC5875350

[mcn13092-bib-0037] Martin, J. A. , Hamilton, B. E. , Osterman, M. J. , Driscoll, A. K. , & Mathews, T. J. (2017). Births: Final data for 2015. National Vital Statistics Reports, 66(1), 1 https://www.cdc.gov/nchs/data/nvsr/nvsr66/nvsr66_01_tables.pdf 28135188

[mcn13092-bib-0038] Maryland Department of Health and Mental Hygiene . (2012). Maryland hospital breastfeeding policy recommendations. Retrieved from https://phpa.health.maryland.gov/wic/Documents/MarylandHospitalBreastfeedingPolicyRecommendations.pdf

[mcn13092-bib-0039] Massachusetts General Hospital . (n.d.). Elective delivery. Retrieved from http://qualityandsafety.massgeneral.org/measures/linemeasurement.aspx?id=923

[mcn13092-bib-0040] Michigan Department of Health and Human Services . (2017). State of Michigan breastfeeding plan 2017–2019. Retrieved from https://www.michigan.gov/documents/infantmortality/Breastfeeding_State_Plan_602456_7.pdf

[mcn13092-bib-0041] Minnesota Breastfeeding Coalition . (2016). Minnesota's progress towards baby friendly hospital designation: 2016 Infant feeding practices survey. Retrieved from https://mnbfc.files.wordpress.com/2013/03/hospital-survey-results_lroberts.pdf

[mcn13092-bib-0042] Minnesota Department of Health . (2018). Maternity care practices and breastfeeding in Minnesota. Retrieved from https://www.health.state.mn.us/docs/people/wic/localagency/reports/bf/info/2018maternity.pdf

[mcn13092-bib-0043] Missouri Department of Health & Senior Services . (n.d.). "Show‐Me 5" initiative. Retrieved from https://health.mo.gov/living/families/wic/breastfeeding/healthcare/showme5/

[mcn13092-bib-0044] National Institute for Children's Health Quality . (2015). NICHQ's final report to Indiana. Retrieved from http://www.state.in.us/isdh/files/NICHQ_Final_Report_to_ISDH.pdf

[mcn13092-bib-0045] Nebraska Department of Health and Human Services . (2015). Breastfeeding of Nebraska Infants—A public health priority. Retrieved from http://dhhs.ne.gov/Documents/MCH%20Breastfeeding%20Assessment%202015.pdf

[mcn13092-bib-0046] Nelson, J. M. , Li, R. , & Perrine, C. G. (2015). Trends of US hospitals distributing infant formula packs to breastfeeding mothers, 2007 to 2013. Pediatrics, 135(6), 1051–1056. 10.1542/peds.2015-0093 26009631 PMC4557614

[mcn13092-bib-0047] Nelson, J. M. , Perrine, C. G. , Freedman, D. S. , Williams, L. , Morrow, B. , Smith, R. A. , & Dee, D. L. (2018). Infant feeding‐related maternity care practices and maternal report of breastfeeding outcomes. Birth, 45(4), 424–431. 10.1111/birt.12337 29411887 PMC9462415

[mcn13092-bib-0048] Nelson, J. M. , Perrine, C. G. , Scanlon, K. S. , & Li, R. (2016). Provision of non‐breast milk supplements to healthy breastfed newborns in US hospitals, 2009 to 2013. Maternal and Child Health Journal, 20(11), 2228–2232. 10.1007/s10995-016-2095-9 27439419 PMC9330174

[mcn13092-bib-0049] Oregon Health Authority . (2017). Achievable goals, infinite rewards breastfeeding in Oregon: 2017. Retrieved from https://www.oregon.gov/oha/ph/healthypeoplefamilies/babies/breastfeeding/documents/bf-whitepaper-2017.pdf

[mcn13092-bib-0050] Patterson, J. A. , Keuler, N. S. , & Olson, B. H. (2018). The effect of maternity practices on exclusive breastfeeding rates in US hospitals. Matern Child Nutr, 15, e12670 10.1111/mcn.12670 30182474 PMC7199031

[mcn13092-bib-0051] Pennsylvania Department of Health . (n.d.). The Keystone 10: A breastfeeding quality improvement initiative for Pennsylvania birthing hospitals and centers. Retrieved from https://www.health.pa.gov/topics/Documents/Programs/Infant%20and%20Children%20Health/1-18_The%20Keystone-%20Initiative_BFH.pdf

[mcn13092-bib-0052] Perrin, M. T. (2018). Donor human milk and fortifier use in United States level 2, 3, and 4 neonatal care hospitals. Journal of Pediatric Gastroenterology and Nutrition, 66(4), 664–669. 10.1097/MPG.0000000000001790 29045350

[mcn13092-bib-0053] Perrine, C. G. , Galuska, D. A. , Dohack, J. L. , Shealy, K. R. , Murphy, P. E. , Grummer‐Strawn, L. M. , & Scanlon, K. S. (2015). Vital signs: Improvements in maternity care policies and practices that support breastfeeding—United States, 2007–2013. MMWR Morb Mortal Wkly Rep, 64(39), 1112–1117. 10.15585/mmwr.mm6439a5 26447527

[mcn13092-bib-0054] Perrine, C. G. , & Scanlon, K. S. (2013). Prevalence of use of human milk in US advanced care neonatal units. Pediatrics, 131(6), 1066–1071. 10.1542/peds.2012-3823 23669517 PMC4535053

[mcn13092-bib-0055] Perrine, C. G. , Scanlon, K. S. , Li, R. , Odom, E. , & Grummer‐Strawn, L. M. (2012). Baby‐friendly hospital practices and meeting exclusive breastfeeding intention. Pediatrics, 130(1), 54–60. 10.1542/peds.2011-3633 22665406 PMC4537174

[mcn13092-bib-0056] Piovanetti, Y. , Calderon, C. , & Castaner, G. (2015). Working for equity in breastfeeding in the maternity services of Puerto Rican hospitals. American Academy of Pediatrics National Conference & Exhibition. Retrieved from https://aap.confex.com/aap/2015/webprogrampress/Paper29421.html

[mcn13092-bib-0057] Preer, G. , Pisegna, J. M. , Cook, J. T. , Henri, A. M. , & Philipp, B. L. (2013). Delaying the bath and in‐hospital breastfeeding rates. Breastfeeding Medicine, 8(6), 485–490. 10.1089/bfm.2012.0158 23635002

[mcn13092-bib-0058] Rosenberg, K. D. , Stull, J. D. , Adler, M. R. , Kasehagen, L. J. , & Crivelli‐Kovach, A. (2008). Impact of hospital policies on breastfeeding outcomes. Breastfeeding Medicine, 3(2), 110–116. 10.1089/bfm.2007.0039 18563999

[mcn13092-bib-0059] RWJF Center for Health Policy at the University of New Mexico . (2016). A review of extant data and data sources related to breastfeeding in New Mexico and recommendations to improve breastfeeding data in the state. Retrieved from https://healthpolicy.unm.edu/sites/default/files/Kellogg%20BF%20Final%20Draft%20-%20Formatted%2012.5.16.pdf

[mcn13092-bib-0060] South Peninsula Hospital . (2016). Birthing center scores high marks from CDC. Retrieved from https://www.sphosp.org/whats-new/south-peninsula-hospital-birthing-center-scores-high-marks-cdc/

[mcn13092-bib-0061] Stockton, J . (n.d.). Increasing access to breastfeeding friendly hospitals: The Iowa experience. Retrieved from https://www.tfah.org/story/increasing-access-to-breastfeeding-friendly-hospitals-the-iowa-experience/

[mcn13092-bib-0062] Substance Abuse and Mental Health Services Administration . (2019). Status report on protecting our infants act implementation plan. Retrieved from https://aspe.hhs.gov/system/files/pdf/260891/POIA.pdf

[mcn13092-bib-0063] Texas Department of State Health Services . (2012). Texas ten step star achiever training toolkit. Retrieved from http://texastenstep.org/starachiever‐texastenstep/Star_Achiever_Ten_Step_Modules/resources‐and‐tools/docs/Texas%20Ten%20Step%20Star%20Achiever%20Training%20ToolKit_Entire%20Toolkit.pdf

[mcn13092-bib-0064] US Department of Health and Human Services . (2011). Executive summary: The surgeon general's call to action to support breastfeeding. Breastfeeding Medicine, 6(1), 3–5. 10.1089/bfm.2011.9996 21332369

[mcn13092-bib-0065] United Health Foundation . (2016). America's health rankings® health of women and children report. Retrieved from https://assets.americashealthrankings.org/app/uploads/hwc-fullreport_v2.pdf

[mcn13092-bib-0066] Whatcom County Health Department . (2011). Whatcom county maternity care practices assessment. Retrieved from https://www.whatcomcounty.us/DocumentCenter/View/1500/Whatcom-County-2010-Maternity-Care-Practices-Assessment-PDF

[mcn13092-bib-0067] World Health Organization . (2018). Implementation guidance: Protecting, promoting and supporting breastfeeding in facilities providing maternity and newborn services—The revised baby‐friendly hospital initiative. Retrieved from https://apps.who.int/iris/bitstream/handle/10665/272943/9789241513807-eng.pdf?ua=1 29565522

